# Supplementary material for: PI3KC2α-dependent and VPS34-independent generation of PI3P controls primary cilium-mediated autophagy in response to shear stress
Source: Nat Commun. 2020 Jan 15;11:294. doi: 10.1038/s41467-019-14086-1 (PMC6962367; doi:10.1038/s41467-019-14086-1)
Supplement: Supplementary file 1 — Supplementary Information [file 41467_2019_14086_MOESM1_ESM.pdf]

Supplementary information

PI3KC2α-dependent and VPS34-independent generation of PI3P controls primary cilium-mediated autophagy in response to shear stress

Boukhalfa, Nascimbeni *et al.*

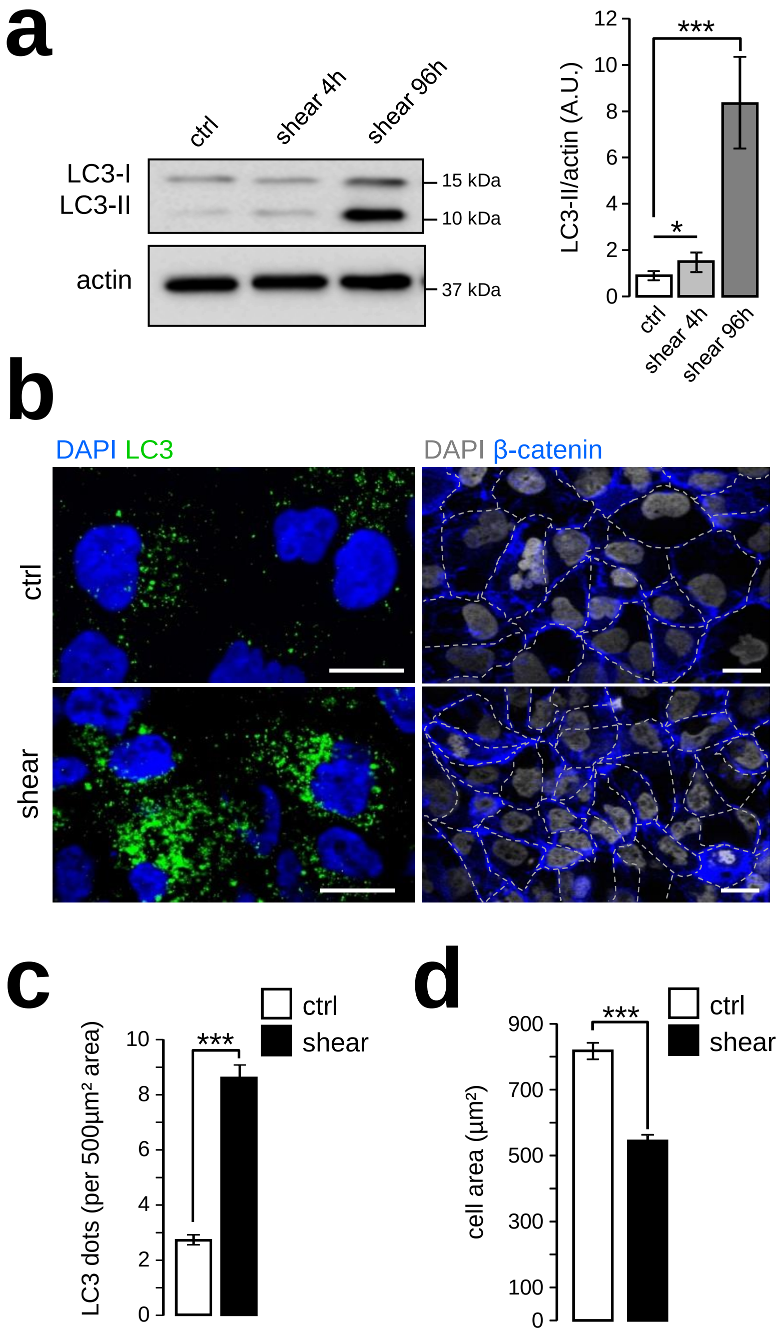

Supplementary Figure 1: Shear stress induces autophagy and regulates cell size in kidney epithelial cells. a Western blot analysis and quantification of LC3-I and LC3-II levels in lysates of polarized HK2 cells subjected to shear stress for 4 and 96 hours, compared to static cultured HK2 cells (ctrl). Bar graph denotes average protein levels normalized to ctrl (mean ± SEM, from 3 independent experiments). LC3 levels were normalized to actin levels (lysate total protein). *p < 0.05, ***p < 0.001 in two-tailed Student's t test. b Representative confocal images of cells subjected to shear stress (96 hours), compared to static cultured cells (ctrl, 96 hours), immunostained for LC3 and -catenin. Dashed white lines are used to better show the cell boundary. Scale bar, 10µm. c, d Bar graphs indicate average number of LC3 puncta per 500 µm2 cellular area and average cell area in cells subjected to shear stress for 96 hours, compared to static condition (ctrl, 96 hours) (mean ± SEM, N = 70 cells, from 3 independent experiments). ***p < 0.001 in two-tailed Student's t test.

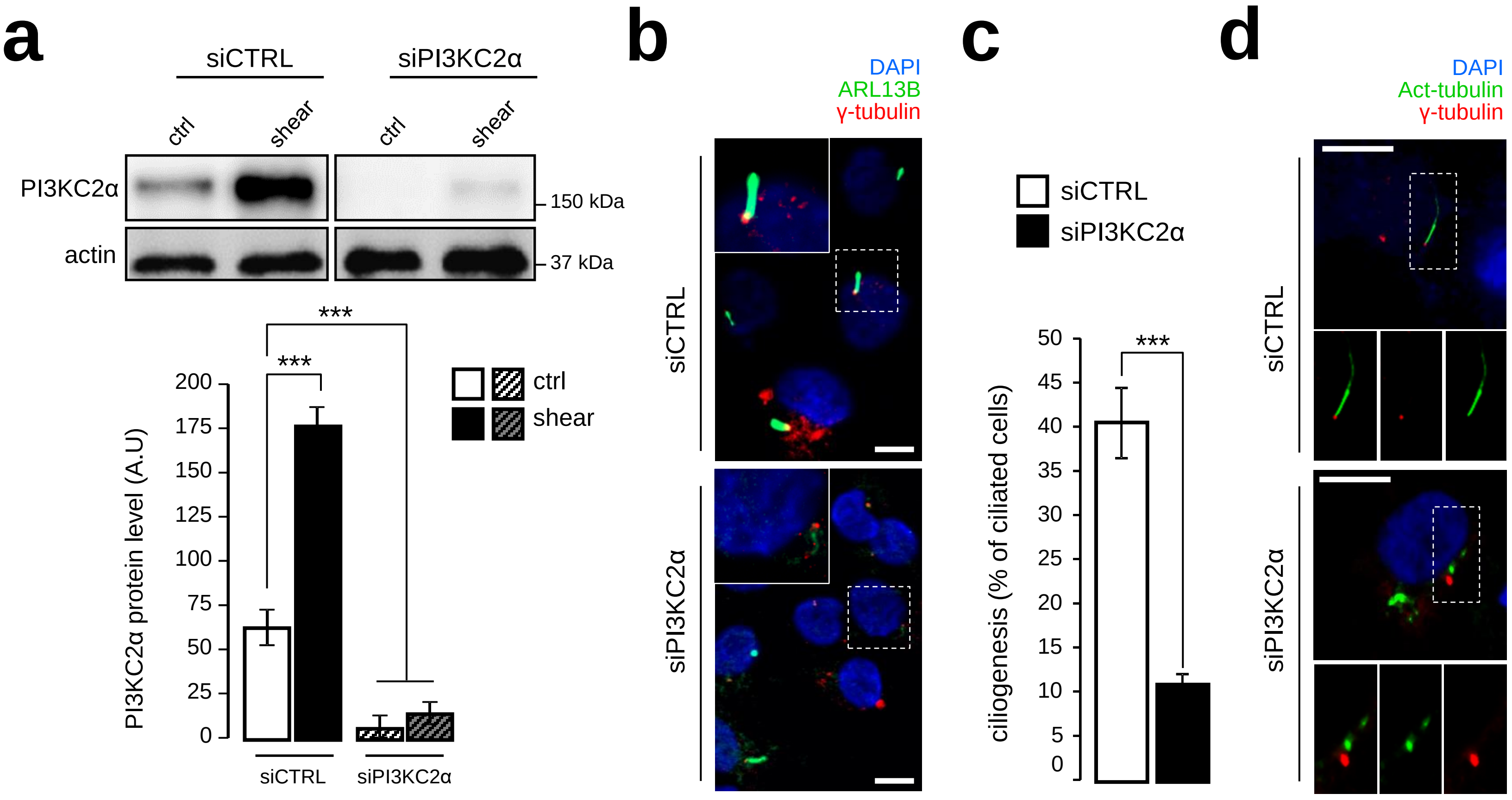

Supplementary Figure 2: PI3KC2α knockdown affects ciliogenesis. a Western blot analysis and quantification of PI3KC2α levels in lysates of HK2 cells (siCTRL), compared to PI3KC2α knocked down cells (siPI3KC2α), upon static (ctrl, 96 hours) and shear stress (96 hours) conditions. Bar graph denotes average protein levels normalized to actin (mean ± SEM, from X independent experiments). ***p < 0.001 in two-tailed Student's t test. b, c Representative confocal images of ciliogenesis (percentage of ciliated cells) in HK2 cells (siCTRL), compared to PI3KC2α knocked down cells (siPI3KC2α), by immunostaining of ARL13B, □-tubulin and DAPI, showing decreased ciliogenesis at the PC in the latter ones, calculated as a percentage of ciliated cells (mean ± SEM, N = 50 cells, from 3 independent experiments). ***p < 0.001 in two-tailed Student's t test. Scale bar, 10µm. d Representative confocal images of HK2 cells (siCTRL), compared to PI3KC2α knocked down cells (siPI3KC2α), by immunostaining of acetylated tubulin (Act-tubulin), □-tubulin and DAPI. Scale bar, 10µm.

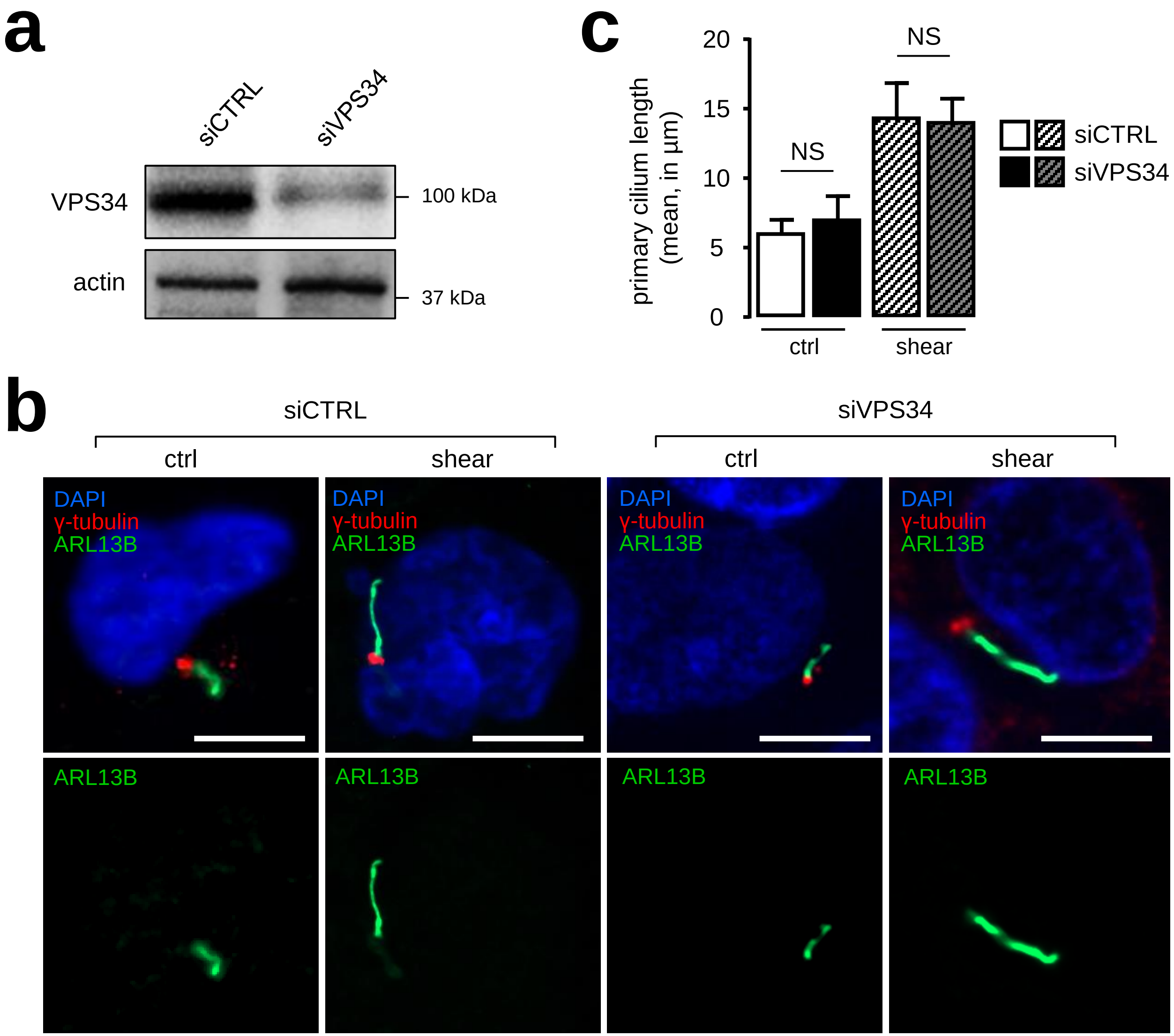

Supplementary Figure 3: PI3KCIII/VPS34 knockdown has no effect on ciliogenesis. a Western blot analysis of VPS34 levels in lysates of HK2 cells (siCTRL), compared to VPS34 knocked down cells (siVPS34), upon shear stress (96 hours) conditions. b, c Representative confocal images of HK2 cells (siCTRL), compared to VPS34 knocked down cells (siVPS34), by immunostaining of ARL13B, □-tubulin and DAPI, showing no effect on primary cilium length (mean ± SEM, N = 50 cells, from 3 independent experiments). ***$p < 0.001$ in two-tailed Student's t test. Scale bar, 10µm.

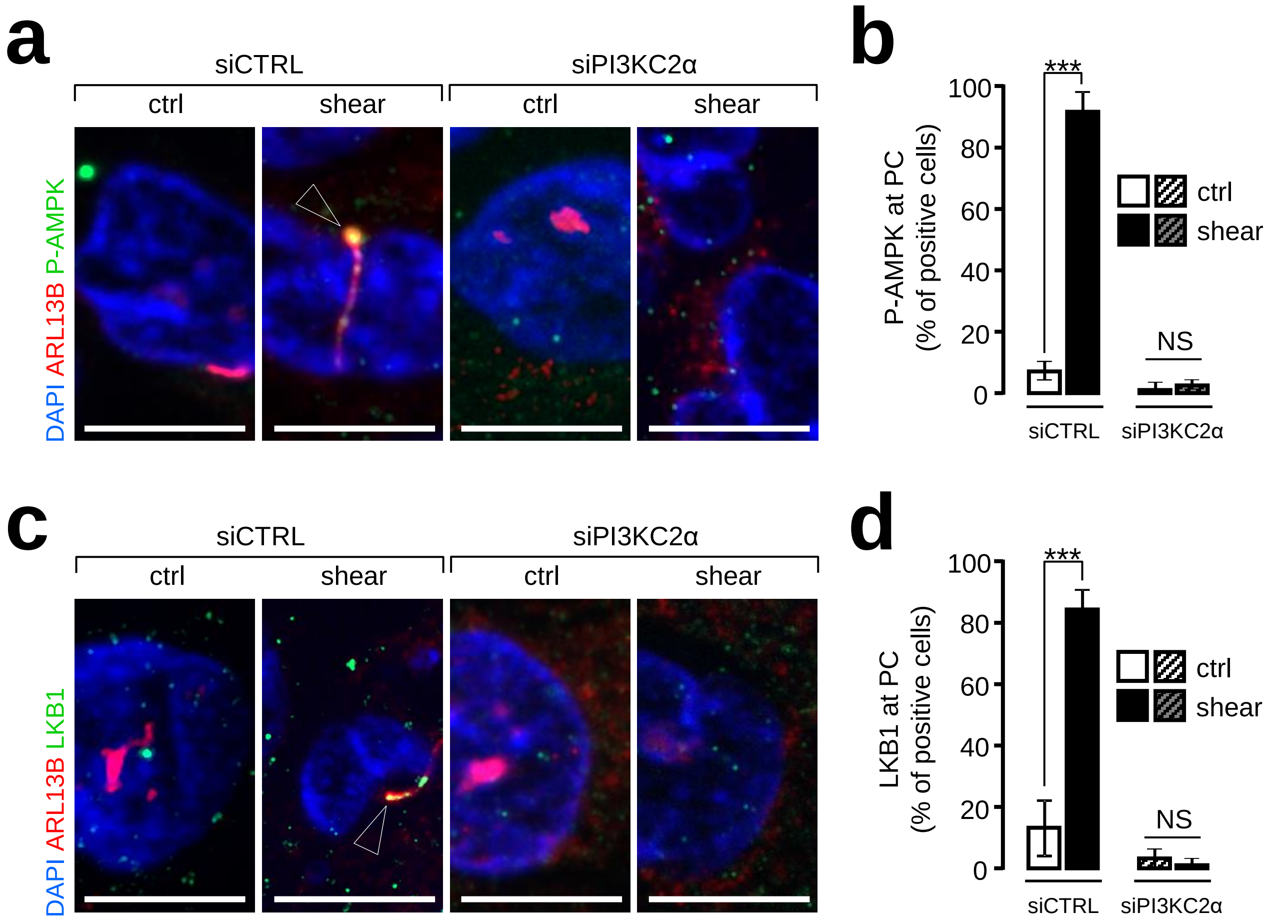

Supplementary Figure 4: PI3KC2α knockdown affects AMPK/LKB1 signalling. a, b representative confocal images and related quantification of TYR172-phospho-AMPK (P-AMPK) recruitment to PC area in HK2 cells (siCTRL), compared to PI3KC2α knocked down cells (siPI3KC2α), by immunostaining of ARL13B, P-AMPK and DAPI, showing recruitment of P-AMPK in shear stress conditions in siCTRL but not in siPI3KC2α cells (mean ± SEM, N = 40 cells, from 3 independent experiments). ***p < 0.001 in two-tailed Student's t test. Scale bar, 10μm. c, d, representative confocal images and related quantification of LKB1 recruitment to PC area in HK2 cells (siCTRL), compared to PI3KC2α knocked down cells (siPI3KC2α), by immunostaining of ARL13B, LKB1 and DAPI, showing recruitment of LKB1 in shear stress conditions in siCTRL but not in siPI3KC2α cells (mean ± SEM, N = 40 cells, from 3 independent experiments). ***p < 0.001 in two-tailed Student's t test. Scale bar, 10μm.

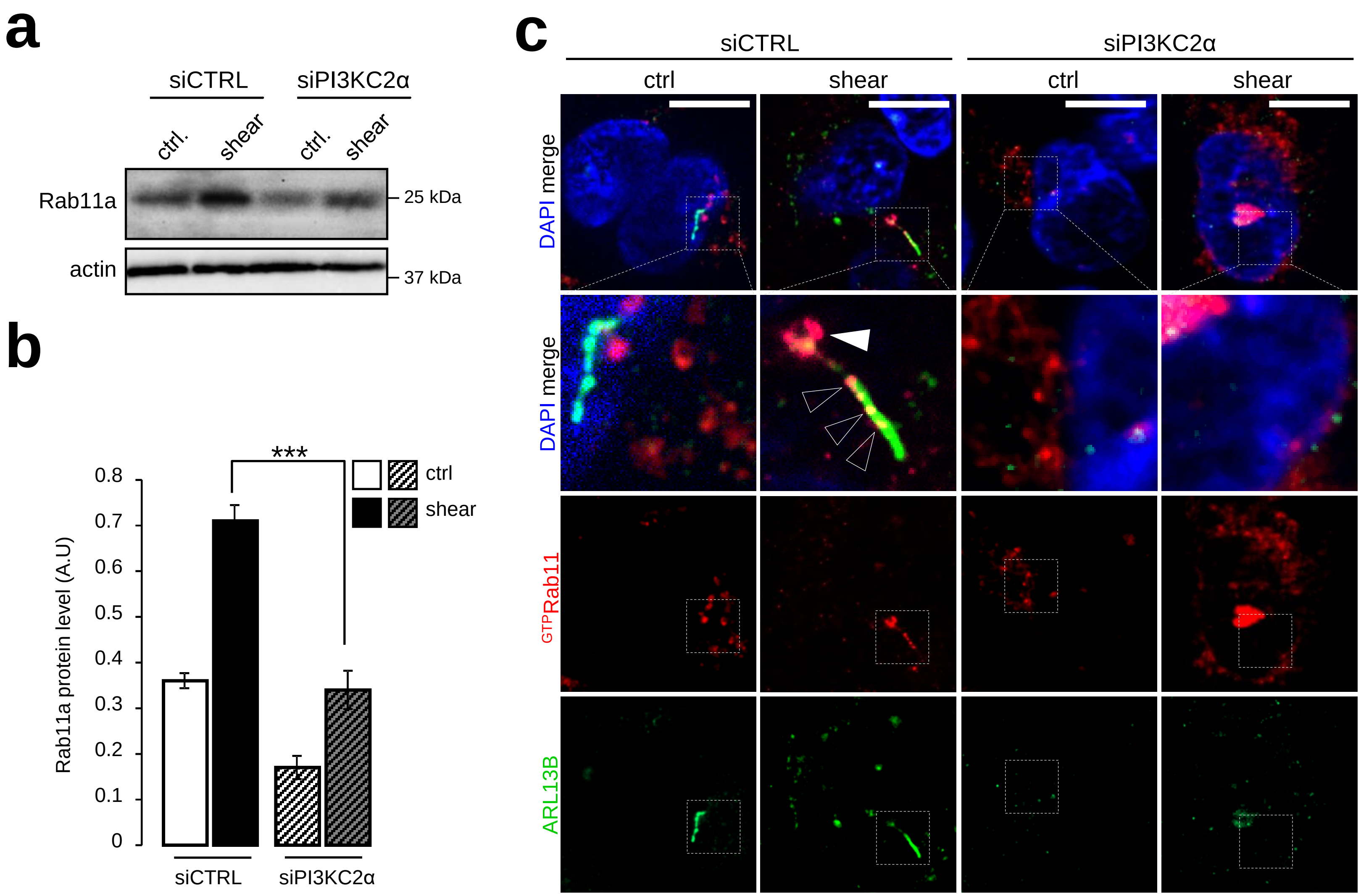

Supplementary Figure 5: Shear stress induces PI3KC2α-dependent Rab11a activation at the PC. a, b Western blot analysis and quantification of Rab11a levels in lysates of HK2 cells (siCTRL), compared to PI3KC2α knocked down cells (siPI3KC2α), upon static (ctrl) and shear stress (96 hours) conditions. Bar graph denotes average protein levels normalized to actin (mean ± SEM, from 3 independent experiments). ***$p < 0.001$ in two-tailed Student's t test. c Representative confocal images of HK2 cells (siCTRL), compared to PI3KC2α knocked down cells (siPI3KC2α), upon static (ctrl, 96 hours) and shear stress (96 hours) conditions, immunostained for GTP bound form of Rab11 (GTPRab11), ARL13B and DAPI (N = 60 cells, from 3 independent experiments). White arrowheads indicate active GTP-bound Rab11 at the basal body of PC and empty arrowheads indicate active GTP-bound Rab11 at the axoneme of PC. Scale bar, 10µm.

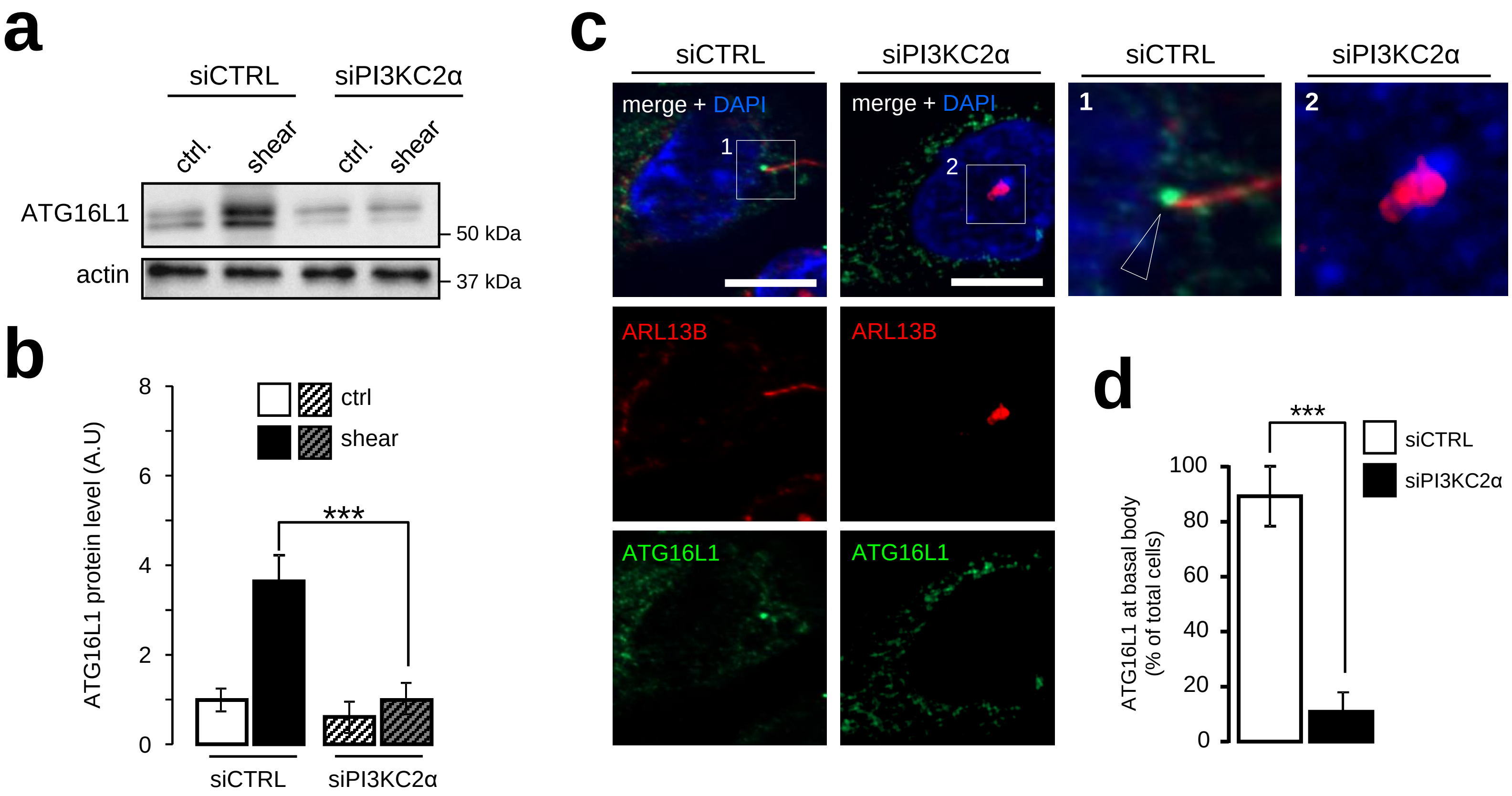

Supplementary Figure 6: Shear stress induces PI3KC2α dependent ATG16L1 recruitment at the PC. a, b Western blot analysis and quantification of ATG16L1 levels in lysates of HK2 cells (siCTRL), compared to PI3KC2α knocked down cells (siPI3KC2α), upon static (ctrl) and shear stress (96 hours) conditions. Bar graph denotes average protein levels normalized to actin (mean ± SEM, from 3 independent experiments). ***p < 0.001 in two-tailed Student's t test. c, d Representative confocal acquisitions, and quantifications (d), of HK2 cells (siCTRL), compared to PI3KC2α knocked down cells (siPI3KC2α), upon static (ctrl, 96 hours) and shear stress (96 hours) conditions, immunostained for ATG16L1, ARL13B and DAPI (N = 60 cells, from 3 independent experiments). Arrowheads indicate ATG16L1 recruitment at the basal body of PC. Scale bar, 10μm.

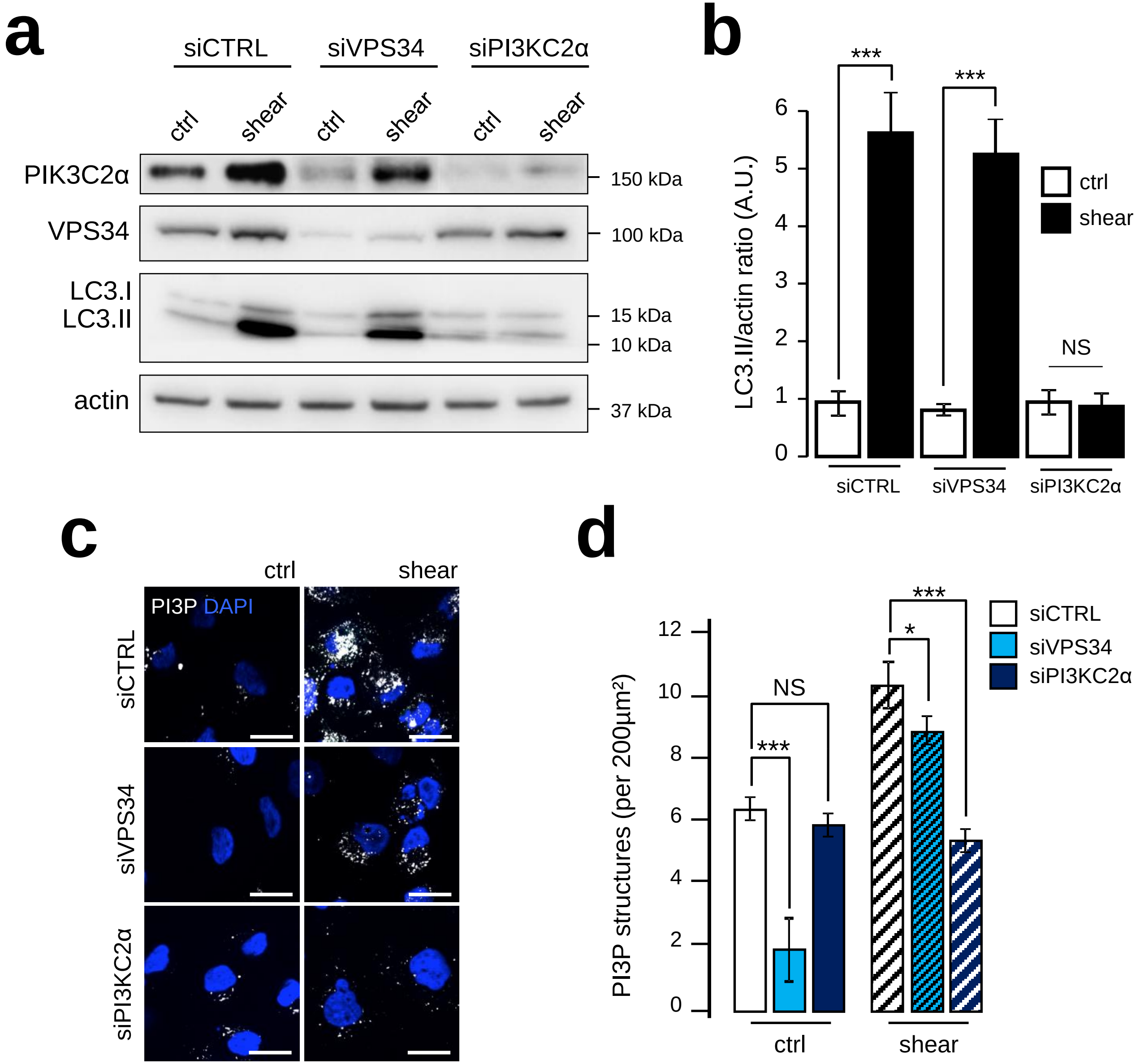

Supplementary Figure 7: Shear stress-induced PI3P synthesis and autophagy depend on PI3KC2α but not on VPS34. a, b Western blot analysis and quantification of PI3KC2α, VPS34, LC3-I and LC3-II levels in lysates of HK2 cells (siCTRL), compared to PI3KC2α knocked down cells (siPI3KC2α), upon static (ctrl, 96 hours) and shear stress (96 hours) conditions. Bar graph denotes average LC3-II protein levels normalized to actin and compared to ctrl (mean ± SEM, from 3 independent experiments). ***$p < 0.001$ in two-tailed Student's t test. c, d Representative confocal images and related quantification of PI3P-positive structures in HK2 cells (siCTRL), compared to PI3KC2α knocked down cells (siPI3KC2α), upon static (ctrl, 96 hours) and shear stress (96 hours) conditions, immunostained for total PI3P-positive intracellular structures and DAPI (mean ± SEM, N = 60 cells, from 3 independent experiments). Bar graph denotes average number of PI3P-positive structures per 200 µm2 area (mean ± SEM, N = 60, from 3 independent experiments). ***$p < 0.001$ in two-tailed Student's t test. Scale bar, 10µm.

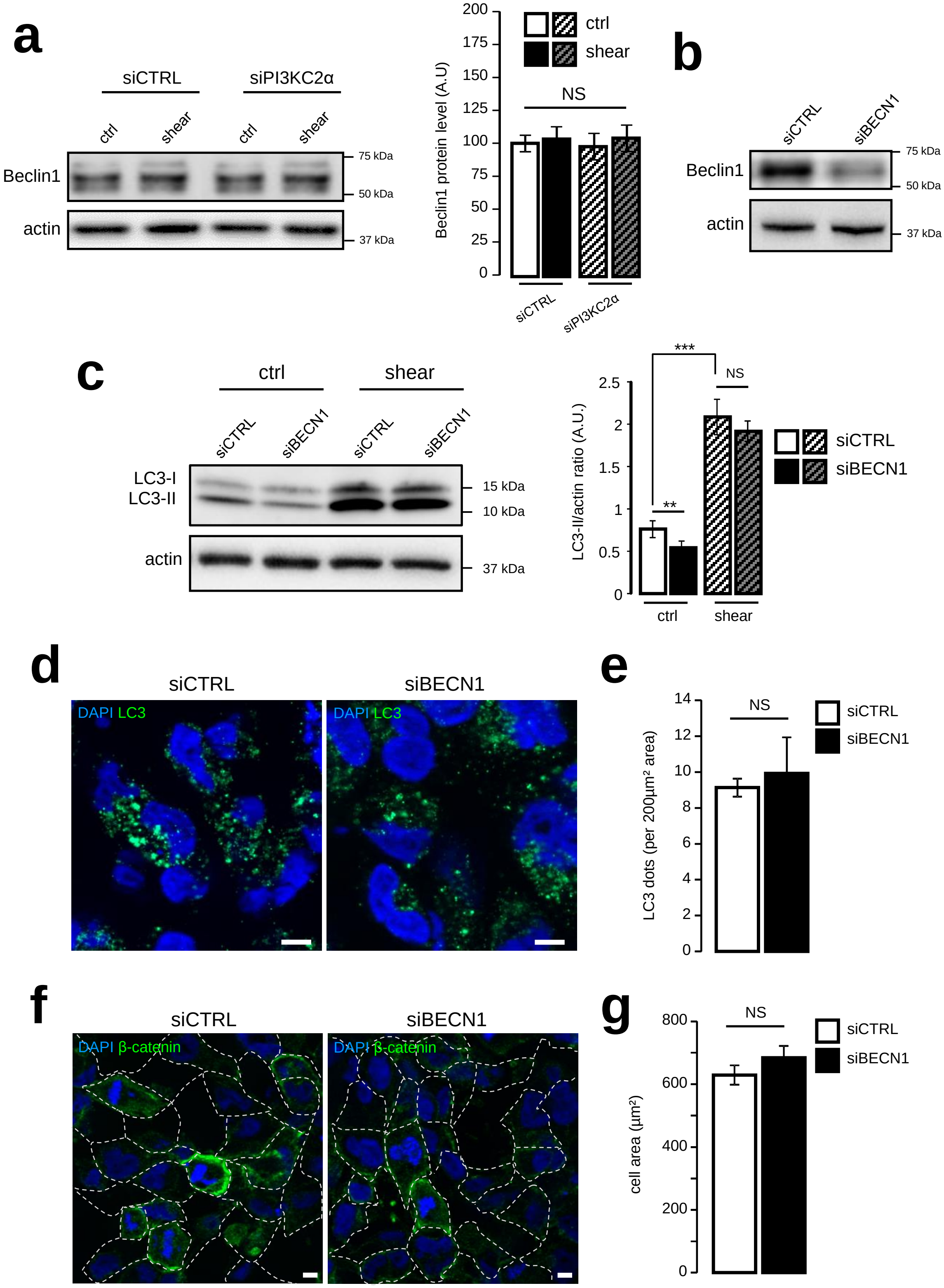

Supplementary Figure 8: Shear stress-induced autophagy is independent of Beclin1. a Western blot analysis and quantification of Beclin1 and actin levels in lysates of HK2 cells (siCTRL), compared to PI3KC2α knocked down cells (siPI3KC2α), upon static (ctrl, 96 hours) and shear stress (96 hours) conditions. Bar graph denotes average Beclin1 protein levels normalized to actin and compared to ctrl (mean ± SEM, from 3 independent experiments). b Western blot representative picture of Beclin1 siRNA (siBECN1) efficiency in HK2 cells. Bar graph denotes average Beclin1 protein levels normalized to actin and compared to ctrl (mean ± SEM, from 3 independent experiments). c Western blot analysis and quantification of LC3.I, LC3.II and actin levels in lysates of HK2 cells (siCTRL), compared to Beclin1 knocked down cells (siBECN1), upon static (ctrl, 96 hours) and shear stress (96 hours) conditions. Bar graph denotes average LC3.II protein levels normalized to actin and compared to ctrl (mean ± SEM, from 3 independent experiments). **$p < 0.05$ in two-tailed Student's t test d, e, f, g Representative confocal images and related quantification of HK2 cells (siCTRL), compared to Beclin1 knocked down cells (siBECN1), upon shear stress (96 hours) conditions, immunostained for LC3 (in d) and β-catenin (in f) and DAPI (mean ± SEM, N = 60 cells, from 3 independent experiments). Bar graph denotes average number of LC3 puncta (in e) per 200 µm2 area (mean ± SEM, N = 60, from 3 independent experiments) and cell area (mean ± SEM, N = 40, from 3 independent experiments). ***$p < 0.001$ in two-tailed Student's t test. Scale bars, 10µm.

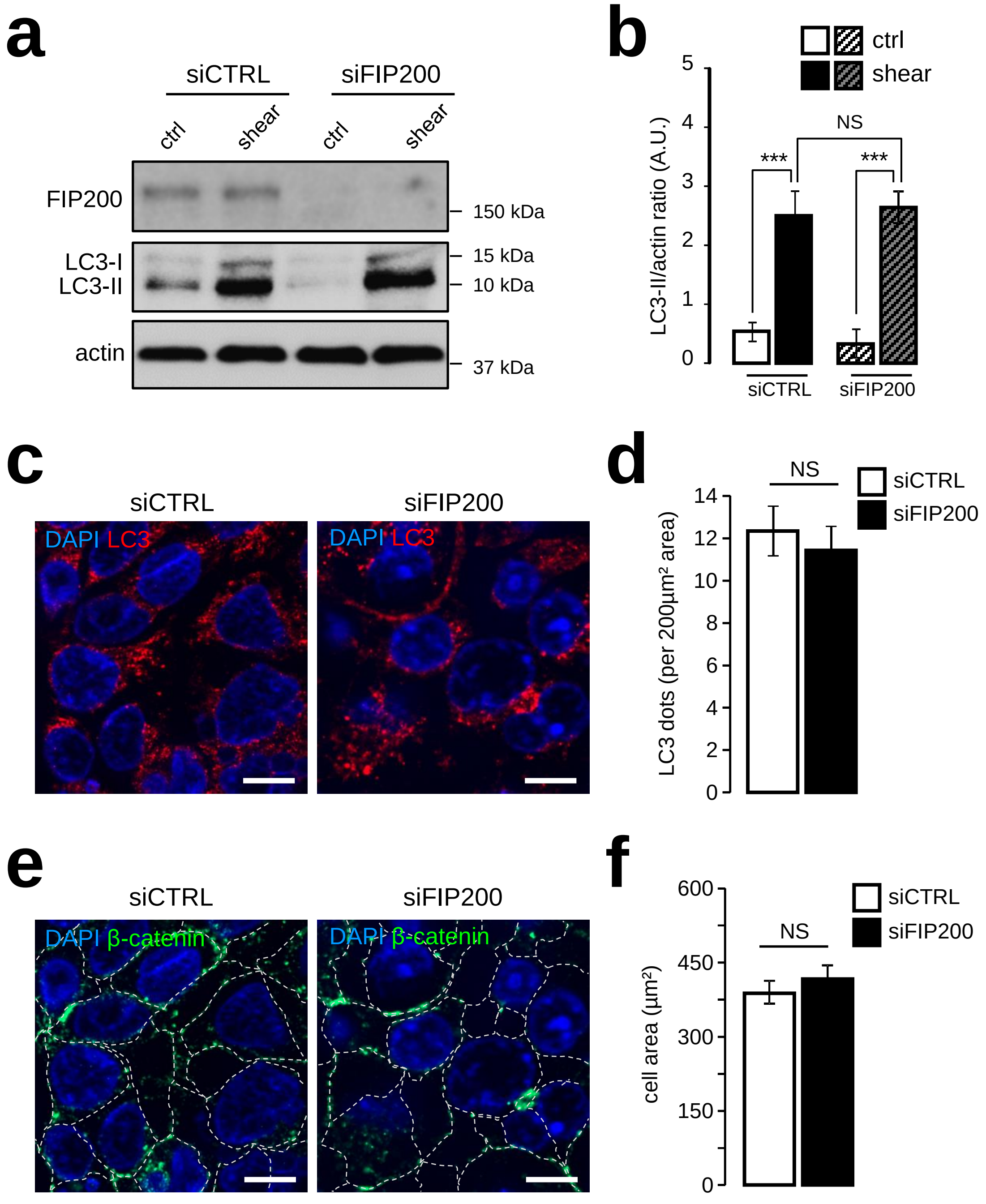

Supplementary Figure 9: Shear stress-induced autophagy is independent of FIP200. a, b Western blot analysis and quantification of FIP200, LC3 and actin levels in lysates of HK2 cells (siCTRL), compared to FIP200 knocked down cells (siFIP200), upon static (ctrl, 96 hours) and shear stress (96 hours) conditions. Bar graph (b) denotes average FIP200 protein levels normalized to actin and compared to ctrl (mean ± SEM, from 3 independent experiments). ***p < 0.05 in two-tailed Student's t test. c, d, e, f Representative confocal acquisitions and related quantifications (d and f) of HK2 cells (siCTRL), compared to FIP200 knocked down cells (siFIP200), upon shear stress (96 hours) conditions, immunostained for LC3 (c) and β-catenin (e) and DAPI (mean ± SEM, N = 60 cells, from 3 independent experiments). Bar graph denotes average number of LC3 puncta (d) per 200 µm2 area (mean ± SEM, N = 60, from 3 independent experiments) and cell area (e, mean ± SEM, N = 40, from 3 independent experiments). ***p < 0.001 in two-tailed Student's t test. Scale bars, 10µm.

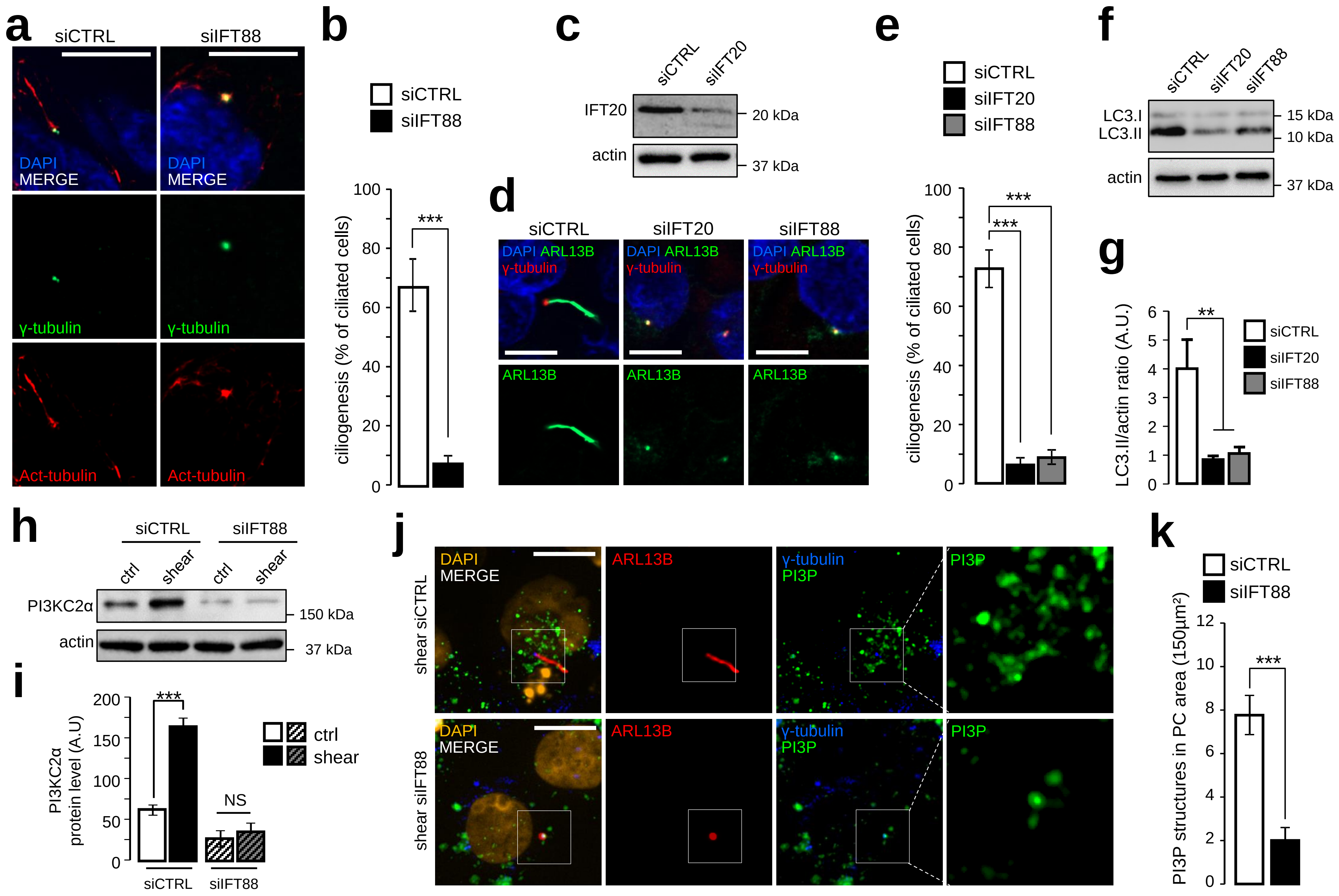

Supplementary Figure 10: characterisation of IFT88 knockdown HK2 cells. a, b Representative confocal analyses (ARL13B, γ-tubulin and DAPI) of ciliogenesis in HK2 cells (siCTRL), compared to siIFT88 cells, showing decreased ciliogenesis at the PC in siIFT88 cells, calculated as a percentage of ciliated cells (mean ± SEM, N = 50 cells, 3 independent experiments). ***p < 0.001 in two-tailed Student's t test. Scale bar, 10µm. c Western blot analysis of IFT20 and actin levels in lysates of HK2 cells (siCTRL), compared to siITF20 cells, upon shear stress (96 hours) conditions. d, e Representative confocal analyses (ARL13B, γ-tubulin and DAPI) of ciliogenesis in HK2 cells (siCTRL), compared to siIFT88 and siIFT20 cells showing decreased ciliogenesis at the PC in siIFT88 and siIFT20 cells, calculated as a percentage of ciliated cells (mean ± SEM, N = 50 cells, 3 independent experiments). ***p < 0.001 in two-tailed Student's t test. Scale bar, 10µm. f, g Western blot analysis and quantification of LC3 and actin levels in lysates of HK2 cells (siCTRL), compared to IFT88 knocked down cells (siIFT88) and IFT20 knocked down cells (siIFT20) upon shear stress (96 hours) conditions. Bar graph (g) denotes average LC3.II protein levels normalized to actin and compared to ctrl (mean ± SEM, 3 independent experiments). **p < 0.05 in two-tailed Student's t test. h, i Western blot analysis and quantification of PI3KC2α and actin levels in lysates of HK2 cells (siCTRL), compared to siIFT88 cells upon static (ctrl, 96 hours) and shear stress (96 hours) conditions. Bar graph (i) denotes average normalized PI3KC2α protein levels compared to ctrl (mean ± SEM, 3 independent experiments). ***p < 0.05 in two-tailed Student's t test. j Representative confocal acquisition upon shear stress conditions of siIFT88 cells, compared to control cells (siCTRL), immunostained for ARL13B, PI3P (FYVE-GST recombinant peptide), γ-tubulin and DAPI. Scale bar, 10µm. k Quantification of the PI3P-positive structures at the PC area in HK2 cells (siCTRL), compared to siIFT88 cells, upon shear stress (96 hours) conditions (mean ± SEM, N = 60 cells, 3 independent experiments). NS: not significant, ***p < 0.001 in two-tailed Student's t test.

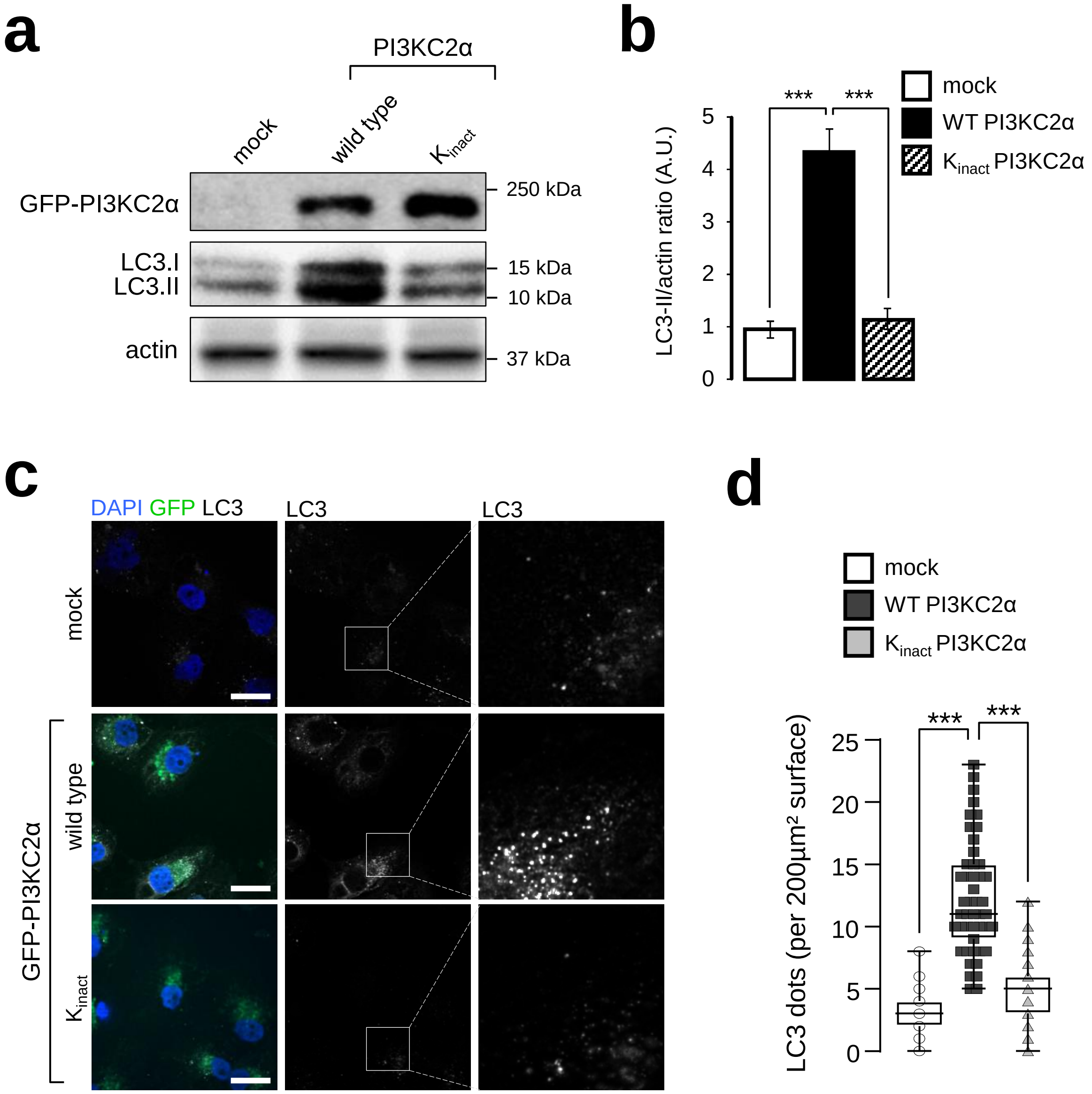

Supplementary Figure 11: the kinase-inactive PI3KC2α mutant does not rescue autophagy in IFT88 knocked-down cells. a, b Western blot analysis of GFP-PI3KC2α variants, LC3 and actin levels in lysates of IFT88 knocked down cells transfected with wild-type PI3KC2α (WT PI3KC2α), kinase-inactive mutant of PI3KC2α (PI3KC2α Kinact) or mock transfected (mock), upon shear stress (96 hours) conditions. Bar graph (b) denotes average LC3.II protein levels normalized to actin and compared to mock condition (mean ± SEM, from 3 independent experiments). ***p < 0.05 in two-tailed Student's t test. c, d Representative confocal acquisition of IFT88 knocked down cells, transfected with wild-type PI3KC2α (WT PI3KC2α), kinase-inactive mutant of PI3KC2α (PI3KC2α Kinact) or mock transfected (mock), upon shear stress (96 hours) conditions, and immunostained for LC3 and DAPI. Scale bar, 10µm. d Bar graphs denote average number of LC3 puncta per 200 µm2 area (mean ± SEM, N = 60, from 3 independent experiments). **p < 0.01, ***p < 0.001 in two-tailed Student's t test.

Figure 1a

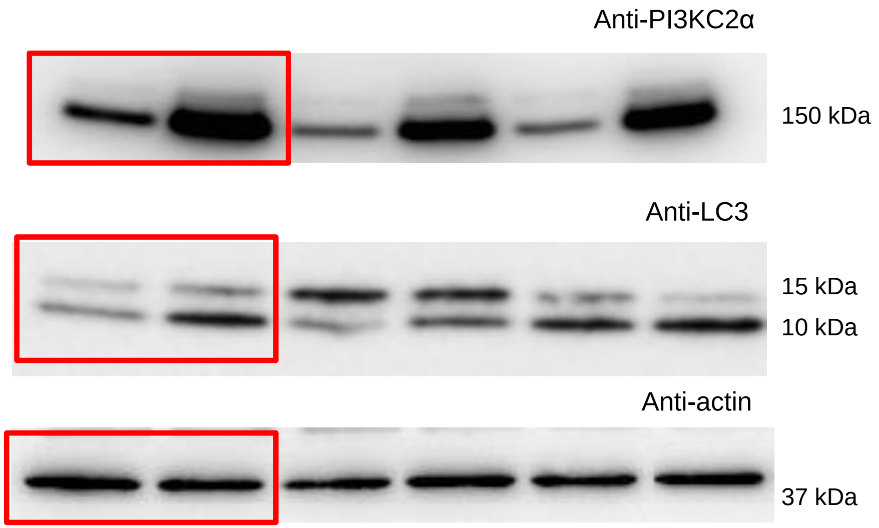

Figure 4b

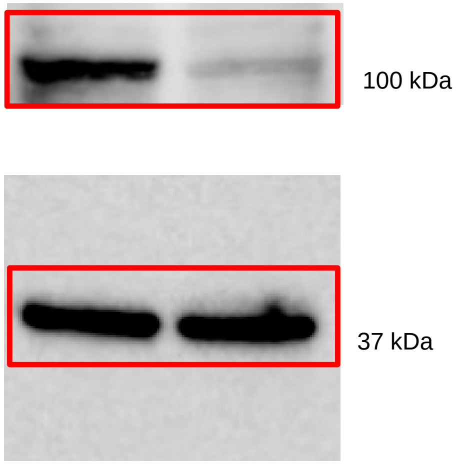

Figure 2d

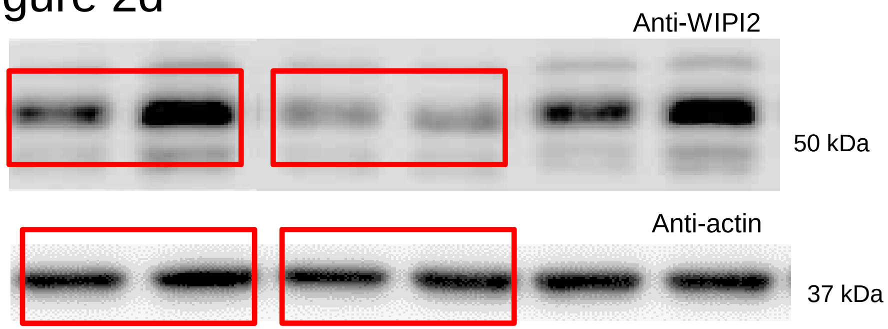

supplementary figure 12 (western blots)
